# A Mental Clinic for London

**Published:** 1908-02-29

**Authors:** 


### A Mental Clinic for London.

Dr. Maudsley's offer to the London County Council of a sum of money towards the establishment of a hospital for the treatment of mental diseases in London draws attention to the fact that no such institution as yet exists in the Metropolis. In other capitals the mental clinic is a reality, doing excellent work and struggling valiantly with the problems of insanity. Here in England we work on somewhat different lines. The student who desires to get an intimate, first hand knowledge of mental disorders is at a serious disadvantage, at least before qualifying. He is allowed, and indeed forced, by the regulations of the various examining bodies, to attend a course of lectures and practical demonstrations at some recognised institution in which mental cases are treated. Such demonstrations are confined, for the most part, to a lecture on a few illustrative cases, preceded or followed by a demonstration on the various methods of certifying. The real routine work of the asylum is practically a closed book to the average student who, under this system, has but little chance of becoming acquainted with the actual treatment of a case of lunacy. Supplemented as the course usually is by regular lectures and regular reading, it yet leaves the student in doubt as to the treatment and diagnosis of many conditions, and if he wishes to perfect himself he can only do so by becoming a clinical assistant or assistant at one of the county asylums or private institutions after qualification. Lunacy is regarded as very much a speciality, and so little of it is seen in the wards of a general hospital that the student gets the impression that it is an equally rare condition outside the precincts of his particular hospital.

The proposed hospital for the treatment of mental diseases, which Dr. Maudsley's munificence has brought within the bounds of an early practical realisation, will supply a very real and pressing need. Everyone must agree with the originator of the scheme which is now receiving the earnest consideration of the Asylums Committee, that there is undoubted scope for such an institution, that has for its primary objects the early treatment of acute cases of mental disorder, the promotion of exact research into the ætiology and pathology of insanity, and the adequate clinical instruction of medical students in mental diseases. These are objects which should have the sympathetic consideration of the London County Council, a body which has already shown itself cognisant of the usefulness of original investigation into mental pathology by the establishment of the Claybury Pathological Laboratory. Lastly, as Dr. Maudsley very justly points out, the establishment of such a hospital as is proposed, an institution in vital touch with the large general hospitals of the Metropolis, will do much to break down "the unfortunate isolation from general medical knowledge and research" in which the study and treatment of insanity remain.

The medical profession, no less than the ratepayers, owes a debt of gratitude to Dr. Maudsley for his timely and thoughtful generosity. Just at present much of our conception of insanity and its treatment is being subjected to criticism, with a view to ultimate amendment and reform. The Royal Commission on the Feeble-minded has done excellent work, and the result of its recommendations, if they are carried out conscientiously, will do much towards improving the system that at present obtains with us.

